# Estimation of the total rectal dose of radical external beam and intracavitary radiotherapy for uterine cervical cancer using the deformable image registration method

**DOI:** 10.1093/jrr/rru127

**Published:** 2015-02-11

**Authors:** Kazuhiko Hayashi, Fumiaki Isohashi, Yuichi Akino, Nobuhide Wakai, Seiji Mabuchi, Osamu Suzuki, Yuji Seo, Yuki Ootani, Iori Sumida, Yasuo Yoshioka, Tadashi Kimura, Kazuhiko Ogawa

**Affiliations:** 1 Department of Radiation Oncology, Osaka University Graduate School of Medicine, Osaka, Japan; 2 Department of Radiology, Suita Tokushukai Hospital, Osaka, Japan; 3 Department of Radiation Oncology, Nara Medical University, Nara, Japan; 4 Department of Obstetrics and Gynecology, Osaka University Graduate School of Medicine, Osaka, Japan

**Keywords:** DIR, cervical cancer, rectal dose, brachytherapy

## Abstract

We adapted the deformable image registration (DIR) technique to accurately calculate the cumulative intracavitary brachytherapy (ICBT) and external beam radiotherapy (EBRT) rectal dose for treating uterine cervical cancer. A total of 14 patients with primary cervical cancer radically treated with ICRT and EBRT were analysed using the Velocity AI^TM^ software. Computed tomography (CT) images were registered, and EBRT and ICBT dose distributions were determined. Cumulative D_2cm_^3^, D_1cm_^3^ and D_0.1cm_^3^ were calculated by simple addition of fractional values or by DIR. The accuracy of DIR was evaluated by means of a virtual phantom mimicking the rectum. The dice similarity coefficient (DSC) was calculated to evaluate rectal contour concordance between CT images before and after DIR. Virtual phantom analysis revealed that the average difference between the DIR-based phantom D_mean_ and the simple phantom D_mean_ was 1.9 ± 2.5 Gy (EQD_2_), and the DIR method included an uncertainty of ∼8.0%. The mean DSC between reference CT and CT was significantly improved after DIR (EBRT: 0.43 vs 0.85, *P* < 0.005; ICBT: 0.60 vs 0.87, *P* < 0.005). The average simple rectal D_2cm_^3^, D_1cm_^3^ and D_0.1cm_^3^ values were 77.6, 81.6 and 91.1 Gy (EQD_2_), respectively; the DIR-based values were 76.2, 79.5 and 87.6 Gy, respectively. The simple addition values were overestimated, on average, by 3.1, 3.7 and 5.5 Gy, respectively, relative to the DIR-based values. In conclusion, the difference between the simple rectal dose–volume histogram (DVH) parameter addition and DIR-based cumulative rectal doses increased with decreasing DVH parameters.

## INTRODUCTION

For radical treatment of cervical cancer, surgery, radiotherapy or chemoradiotherapy is generally used. For radical radiotherapy, both external beam radiotherapy (EBRT) and intracavitary brachytherapy (ICBT) are commonly performed. Recently, 3D image-guided radiotherapy has been widely used in both EBRT and ICRT. The Groupe Européen de Curiethérapie–European Society for Therapeutic Radiology and Oncology (GEC–ESTRO) working group recommended evaluation of 3D dose–volume parameters [1–3]. For organs at risk, reporting the minimum dose in the most irradiated tissue volume is recommended. However, the GYN GEC-ESTRO working group mentioned that, in fractionated brachytherapy, the location of the high-dose region may not be identical for each fraction. Therefore, estimating the cumulative dose by adding dose–volume histogram (DVH) parameters of risk organs for EBRT and ICBT would be a ‘worst case assumption’ [2]. However, it is not easy to estimate the true cumulative dose of EBRT and ICBT because the use of a tandem/ovoid applicator for ICBT changes the position, shape and volume of the organs at risk.

To calculate the cumulative dose accurately, we used the deformable image registration (DIR) method, which computes a voxel-to-voxel map from a reference image to a target image. In concrete terms, after we deformed registered computed tomography (CT) images on the basis of a reference CT image, the dose distribution of the registered CT images was also deformed and the cumulative dose of the risk organs was calculated.

When EBRT and ICBT are performed for primary cervical cancer, the main late toxicity among organs at risk is rectal bleeding. We used the rectal D_2cm_^3^, D_1cm_^3^ and D_0.1cm_^3^ values relevant for rectal bleeding according to the GYN GEC–ESTRO working group recommendations [4]. In this study, we investigated the difference between simple rectal DVH parameter addition and DIR-based cumulative rectal doses.

## MATERIALS AND METHODS

### Virtual phantom study

**Fig. 1.**
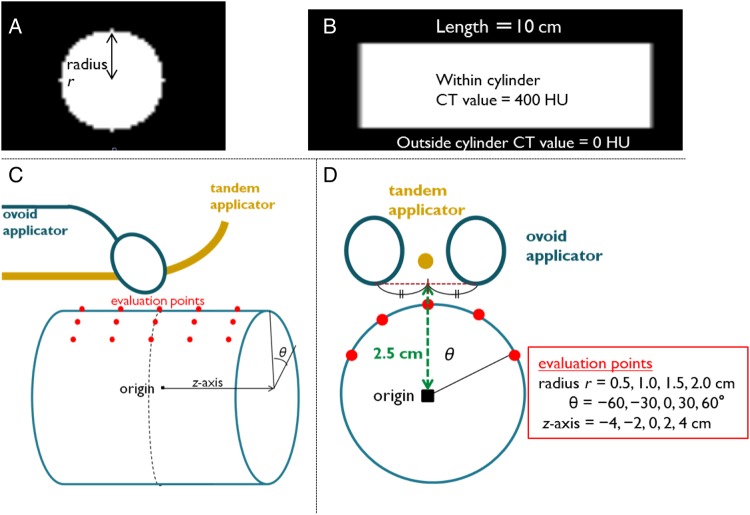
Virtual phantom. Four cylindrical virtual phantoms mimicking the rectum were generated on the CT image set. (**A**) Axial and (**B**) sagittal planes. The radii of the phantoms were 0.5, 1.0, 1.5 and 2.0 cm and the lengths were all 10 cm. (**C**) Schematic diagram of the virtual phantom study and (**D**) a cranial view. The typical dose distributions of ICBT were projected onto the virtual phantom CT images. When the typical dose distribution was generated, virtual tandem and ovoid applicators were set. There were 25 points for dose evaluation located on the surface of the phantom. Abbreviations: CT = computed tomography.

To investigate the validity of the DIR method for ICBT, four cylindrical virtual phantoms mimicking the rectum were generated (Fig. 1A and B) on a void CT image set. The radii of the phantoms were 0.5, 1.0, 1.5 and 2.0 cm. The CT values of the voxels inside and outside the phantom were set to 400 and 0 Hounsfield units (HU), respectively. We selected a representative dose distribution of ICBT (Fig. 1C and D) from dose distributions of patients on whom ICBT was performed. The patient's prescribed dose to point A, which was described by a fixed point 2 cm lateral to the uterine axis and 2 cm above the lateral fornix, was 6.8 Gy/fraction. The typical dose distribution was projected onto the virtual CT images. When the origin, radius (*r*), angle (*θ*) and *z* axis of the virtual phantom were established, as shown in Fig. 1C and D, the patient's prescribed point corresponded to the point (*r*, *θ*, *z*) = (45 mm, 27°, 16 mm) in the coordinates of the virtual phantom. On the basis of the selected typical dose distribution, we established virtual tandem and ovoid applicators. The width of the right and left ovoid applicators was 3 cm. The distances between the tandem applicator and the cylindrical phantoms at radii of 0.5 cm, 1 cm, 1.5 cm and 2 cm were 0.65 cm, 1.15 cm, 1.65 cm and 2.15 cm, respectively. The number was given every 2.5 mm, and number one was defined as a point 7 mm from the tip of the applicator. The pattern of the source arrangement of ovoid applicators was 1, 3, 5 and 7. That of the tandem applicator was 1, 4, 7, 11 and 15. We simulated the pseudo-rectum by varying its radius (*r*) at each fraction: *r* = 1.5, 0.5, 1.0 or 2.0 cm at the first, second, third or fourth ICBT, respectively. We assumed that varying the radii of the cylinders would represent deformation of the rectum by faeces or gas between the fractions of ICBT. The mean doses of the four fractions were evaluated at 25 points located on the surface of the phantom. When the radius of the pseudo-rectum was changed, these points moved radially and did not move along the longitudinal direction. The mean dose (simple phantom D_mean_) was calculated for each evaluated point by simply averaging the dose of the four treatment fractions.

The mean dose at the evaluation points was also calculated using DIR for the CT images. The CT image set of the first fraction containing a phantom with a radius of 1.5 cm was considered to be the reference CT image set, and the image sets of the other three fractions were deformed toward the reference images using Velocity AI™ software (version 2.7; Velocity Medical Solutions, Atlanta, GA). Dose distributions were also deformed using the calculated deformation vector fields and were projected onto the reference CT images. The mean doses at the evaluation points (DIR-based phantom D_mean_) were calculated. To evaluate the validity of the DIR method for ICBT, we determined the correlation coefficient and the difference between the DIR-based phantom D_mean_ and the simple phantom D_mean_.

### Patient characteristics

This study was performed in accordance with the guidelines approved by the institutional review board of our institution. A total of 24 patients with primary cervical cancer received definitive EBRT and ICRT at the Osaka University Hospital from April 2012 to July 2013. Ten patients were excluded from this study. Eight of the 10 patients were excluded because the length, shape or insertion of a tandem applicator (anteriorly or posteriorly) was changed for each fraction of ICBT. Two of the 10 patients could not complete chemoradiation; one patient had cancers of the cervix and ovary, and another showed a strong side effect from chemotherapy. Consequently, we retrospectively analysed 14 patients. All patients were diagnosed by palpation, imaging (plain radiography, computed tomography, magnetic resonance imaging or positron emission tomography) and biopsies. The distribution of the TNM classifications was as follows: three patients had cT2bN0M0, three had cT2bN1M0, two had cT2bN1M1, one had cT3bN0M0, three had cT3bN1M0, one had cT3bN1M1 and one had a cT4aN0M0 tumour. The median age of the patients was 61 years (range: 31–78 years).

### Treatment

Of the 14 patients, 11 received concurrent chemoradiation, and the others received radiation alone. The following chemotherapy regimens were used: 6 patients received nedaplatin, 4 received paclitaxel/carboplatin and 1 received cisplatin.

For radiotherapy, both EBRT and ^192^Ir high-dose-rate ICBT were performed for all patients as previously described, with some modification [5]. Whole-pelvis or extended-field EBRT with the 4-field box technique using 10-MV X-rays was followed by ICBT, and additional EBRT with a midline block (width = 4 cm) was delivered through anterior and posterior parallel-opposed portals. ICBT was performed one or two times per week, along with the central shielding EBRT. The irradiated doses are shown in Table 1. For ICBT, we used a set of Fletcher-type metal applicators (Nucletron International BB, Veenendaal, The Netherlands). After the applicators were inserted into the vagina, gauze was packed on the anterior and posterior sides of the applicators and CT images were obtained. We used Oncentra (version 4.1; Nucletron International BB, Veenendaal, The Netherlands) to make a 3D treatment plan on the basis of the CT images. The ICBT dose was prescribed to point A; then, we decreased the dose on the basis of the dose constraints that D_2cm_^3^ of a rectum or bladder does not exceed 7 Gy. The pattern of the source arrangement is shown in Supplementary Table 1. Treatment planning and the dose distribution for EBRT were calculated using XiO (version 4.5; Elekta, Stockholm, Sweden).

**Table 1.** Treatment schedule for cervical cancer

WP	CS (the width = 4 cm)	ICBT	Number of patients
20 Gy/10 fr	30 Gy/15 fr	20.4 Gy/3 fr	6
10 Gy/5 fr	40 Gy/20 fr	27.2 Gy/4 fr	4
10.8 Gy/6 fr	39.6 Gy/22 fr	20.4 Gy/3 fr	1
19.8 Gy/11 fr	30.6 Gy/17 fr	27.2 Gy/4 fr	1
20 Gy/10 fr	30 Gy/15 fr	26.8 Gy/4 fr	1
10.8 Gy/6 fr	39.6 Gy/22 fr	19.6 Gy/3 fr	1

WP = whole-pelvis irradiation, CS = center shielding (midline block for pelvic irradiation), ICBT = intracavitary brachytherapy, fr = fractions.

### Simple rectal DVH parameter addition

We defined the cumulative doses by adding the rectal D_2cm_^3^, D_1cm_^3^ and D_0.1cm_^3^ values of EBRT and ICBT as simple rectal D_2cm_^3^, D_1cm_^3^ and D_0.1cm_^3^ values, respectively. Simple rectal D*_x_*_cm_^3^ (*x* = 2, 1 or 0.1) was defined according to the following formula:

$$\text{Simple}\;\text{rectal}\;{\text{D}}_{\mathrm{xcm}}^{3}={\text{D}}_{\mathrm{xcm}}^{3}(\text{EBRT})+\sum_{i=1}^{n}{\text{D}}_{\mathrm{xcm}}^{3}(\text{ICBT}{)}_{i},$$

where D*_x_*_cm_^3^ (EBRT) and D*_x_*_cm_^3^ (ICBT) represent the dose delivered to a certain volume (*x* cm^3^) by EBRT (whole pelvic irradiation dose, excluding the fractions with central shielding) and ICBT, respectively. For ICBT, the fraction number (*n*) was 3 or 4, depending on the stage of each patient. Every D*_x_*_cm_^3^ was calculated as the biologically equivalent dose in 2-Gy fractions (EQD_2_) according to the linear quadratic model (LQ model) using α/β = 3 [6].

### DIR-based rectal dose calculation

**Fig. 2.**
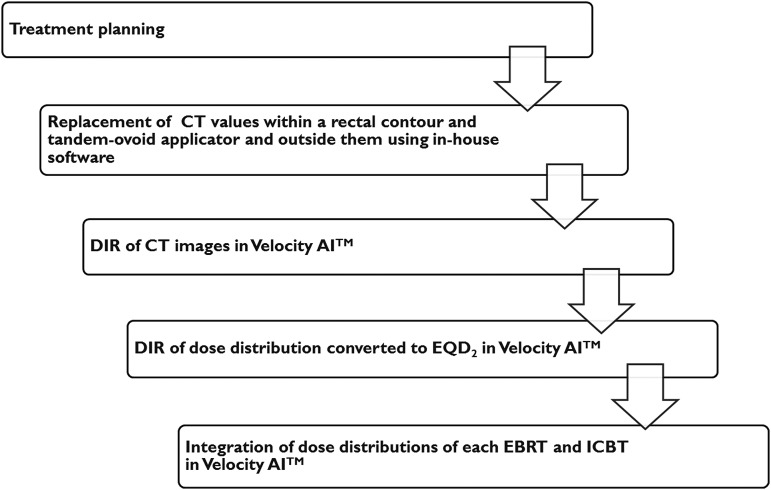
The procedure to calculate the DIR-based rectal dose. Abbreviations: CT = computed tomography, DIR = deformable image registration, EBRT = external beam radiotherapy, ICBT = intracavitary brachytherapy.

**Fig. 3.**
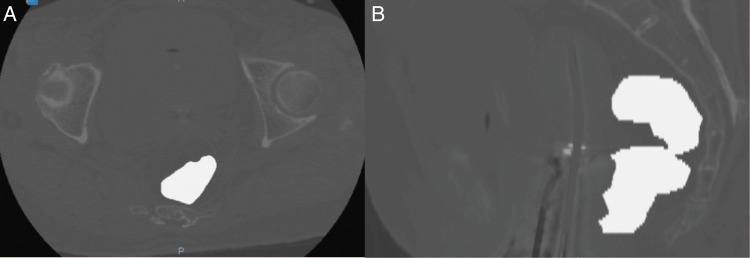
Typical CT images after replacement of internal and external CT values for the rectum and tandem/ovoid applicator. To eliminate the effects of metal artefacts on the CT images, the CT values within a rectal contour or tandem/ovoid applicator were replaced by 400 or 0 HU, respectively, and the CT values outside the rectal contours were multiplied by 0.01. Abbreviations: CT = computed tomography.

**Fig. 4.**
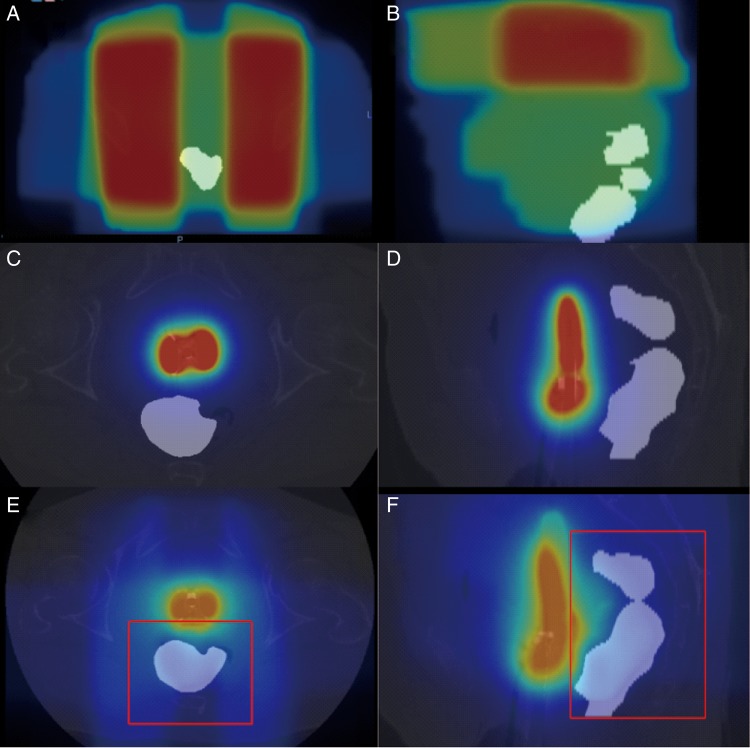
The typical dose distribution projected to CT images before and after DIR. (**A**) The dose distribution of EBRT before DIR from axial view. To make it easy to see, CT values within a rectal contour and tandem/ovoid applicator and outside them were replaced using in-house software; (**B**) sagittal view. (**C**) The dose distribution of ICBT before DIR from the axial view; (**D**) sagittal view. (**E**) The cumulative dose distribution after DIR. An area surrounded by the red line displayed a rectangular parallelepiped volume of interest that included the rectal contours. Abbreviations: CT = computed tomography, DIR = deformable image registration, EBRT = external beam radiotherapy, ICBT = intracavitary brachytherapy.

We defined DIR-based D_2cm_^3^_,_ D_1cm_^3^ and D_0.1cm_^3^ as cumulative D_2cm_^3^_,_ D_1cm_^3^ and D_0.1cm_^3^, estimated on the basis of the DIR method, respectively. The procedure for calculating DIR-based rectal D*_x_*_cm_^3^ (*x* = 2, 1 or 0.1) is shown in Fig. 2. On the CT images for both EBRT and ICBT, rectal contours were delineated. The rectal contour was defined as a solid volume with the range from the anus to the caudal edge of the second sacral vertebra. To eliminate the effects of metal artefacts on the CT images, the CT values within a rectal contour or tandem/ovoid applicator were replaced by 400 or 0 HU, respectively, and the CT values outside the rectal contours were multiplied by 0.01, as shown in Fig. 3 [7]. The CT images and DICOM RT structure files were imported into Velocity AI™ software. We performed rigid registration on the basis of the bone, followed by DIR for faster and accurate registration. The CT images of the first fraction of ICBT were used as a reference CT image set for DIR. DIR was performed inside a rectangular parallelepiped volume of interest (shown in Fig. 4E and F) that included the rectal contours. The dose distribution was rescaled to evaluate the biologically equivalent doses as EQD_2_ according to the linear quadratic model using α/β = 3 [5] and deformed using the deformation vector fields calculated for the CT images. All dose distributions of ICBT and EBRT (whole pelvic irradiation dose, including the fractions with central shielding) were integrated using Velocity AI^TM^ software (shown in Fig. 4). Finally, the DIR-based rectal D*_x_*_cm_^3^ was calculated from the integrated dose distribution. We confirmed that all points of D*_x_*_cm_^3^ by DIR-based calculation existed within the midline block.

### Dice similarity coefficient

The dice similarity coefficient (DSC), defined according to the following formula, was calculated to evaluate the concordance between the CT images before and after deformable registration:

$$\text{DSC }({\text{A}}_{\mathrm{ref}}\;,\text{B})=\text{2 }({\text{A}}_{\mathrm{ref}}\cap \text{B})/({\text{A}}_{\mathrm{ref}}+\text{B}),$$

where A_ref_ and B represent the rectal contours on the reference and evaluated CT image sets, respectively [8]. DSC gave measures of the volumetric overlap between two of the segmented structures and ranged from 0 to 1, where 0 is no alignment between images and 1 is perfect alignment.

### Statistical analysis

We used Student's *t*-test to compare DSCs before and after DIR. Statistical significance was set at a *P*-value of < 0.05. We calculated the Pearson product correlation coefficients for the results of the virtual phantom analyses.

## RESULTS

### Virtual phantom study

**Fig. 5.**
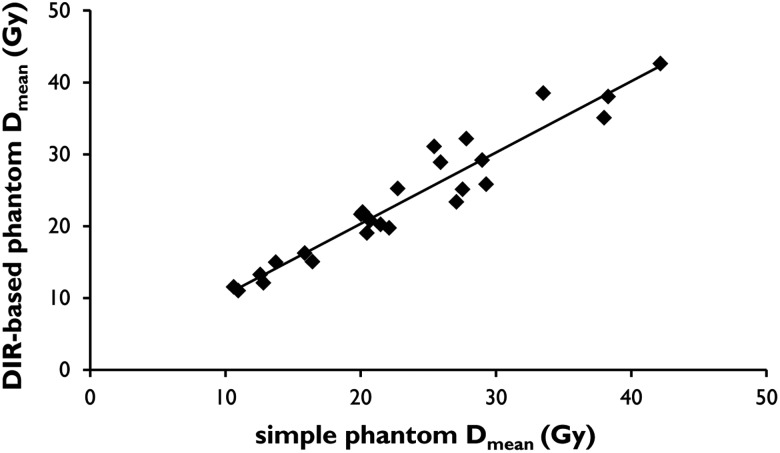
Scatter diagram comparing DIR-based phantom Dmean with simple phantom Dmean. A strong correlation between the DIR-based phantom Dmean and the simple phantom Dmean was observed (correlation coefficient = 0.96). Abbreviations: DIR = deformable image registration.

Figure 5 shows the scatter diagram for the comparison of the DIR-based phantom D_mean_ with the simple phantom D_mean_ evaluated at 25 points. The correlation coefficient was 0.96, which indicated a strong correlation between the two D_mean_ values. The average difference between the DIR-based phantom D_mean_ and the simple phantom D_mean_ was 1.9 ± 2.5 Gy (EQD_2_). The average relative difference was 8.0% ± 5.4% for the comparison of the simple phantom D_mean_. These results revealed that the DIR method included an uncertainty of ∼8.0%.

### Patient study

**Fig. 6.**
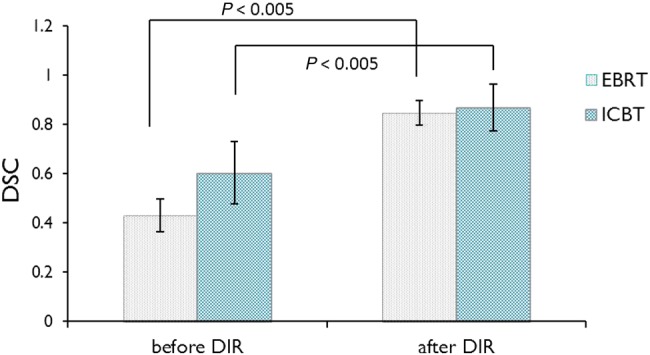
The DSC before and after DIR. The results of the DSC revealed that the DIR method significantly improved the DSC between the reference CT and the CT of EBRT or ICBT from 0.43 to 0.85 and from 0.60 to 0.87, respectively. Abbreviations: DIR = deformable image registration, DSC = Dice similarity coefficient.

DIRs of all treatment plans for the 14 patients (Patient Nos 1 to 14) were performed. We visually checked all images after deformation, and no physiologically implausible deformation was found. We also calculated the DSC to evaluate whether the DIR method was appropriately performed. The results are shown in Fig. 6. The mean DSC of the rectal contours between the reference CT and the CT of EBRT significantly improved from 0.43 before DIR to 0.85 after DIR (*P* < 0.005). Similarly, the value between the reference CT and the CT of other ICBT fractions significantly improved from 0.60 to 0.87 (*P* < 0.005). These results showed that the DIR method worked appropriately for patient study.

**Fig. 7.**
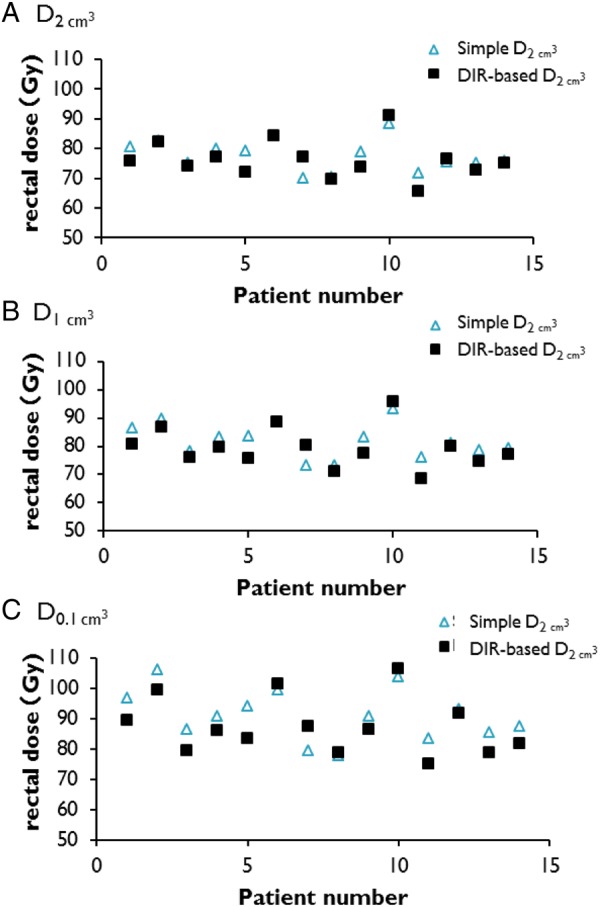
DIR-based or simple rectal D*_x_*_cm_^3^ values for each patient (*x* = 2, 1 or 0.1): (**A**) D_2cm_^3^, (**B**) D_1cm_^3^, (**C**) D_0.1cm_^3^. A triangles and squares expressed simple D*_x_*_cm_^3^ and DIR-based D*_x_*_cm_^3^. These results showed that the simple addition method tended to overestimate rectal D*_x_*_cm_^3^ compared with the DIR-based method. Abbreviations: DIR = deformable image registration.

The DIR-based rectal D*_x_*_cm_^3^ and simple rectal D*_x_*_cm_^3^ values (*x* = 2, 1 or 0.1) were calculated. Figure 7 shows the DIR-based or simple rectal D*_x_*_cm_^3^ values for each patient. These results showed that the simple addition method tended to overestimate rectal D*_x_*_cm_^3^ compared with the DIR-based method. The average simple rectal D_2cm_^3^, D_1cm_^3^ and D_0.1cm_^3^ values were 77.6, 81.6 and 91.1 Gy, respectively, and the DIR-based D_2cm_^3^, D_1cm_^3^ and D_0.1cm_^3^ values were 76.2, 79.5 and 87.6 Gy (EQD_2_), respectively. The mean absolute value of the difference between the DIR-based rectal D_2cm_^3^ and the simple rectal D_2cm_^3^ was 3.1 ± 2.6 Gy (range: 0.1–7.3 Gy), whereas those for D_1cm_^3^ and D_0.1cm_^3^ were 3.7 ± 2.5 Gy and 5.5 ± 3.0 Gy, respectively. The relative differences of D_2cm_^3^, D_1cm_^3^ and D_0.1cm_^3^ in comparison with the DIR-based rectal D*_x_*_cm_^3^ values were 4.1% ± 3.6%, 4.7% ± 3.4% and 6.4% ± 3.7%, respectively. Two patients showed relative differences > 8%.

## DISCUSSION

We applied the DIR method reported by Akino *et al.* to calculate the accumulated rectal dose of multiple treatments in EBRT and ICRT [7]. To verify the validity of registration, we evaluated the accuracy of DIR by means of DSC analysis and virtual phantom analysis. The results of DSC revealed that the DIR method significantly improved the DSC between the reference CT and the CT of EBRT or ICBT from 0.43 to 0.85 and from 0.60 to 0.87, respectively. These findings were consistent with the results reported by Akino *et al.*, who showed that DSC increased from 0.75 to 0.90 by using DIR for rectal contouring of each EBRT fraction for prostate cancer [7]. Considering the use of a tandem/ovoid applicator for ICBT for cervical cancer changes the rectal form more greatly than the use for EBRT for prostate cancer, we concluded that the improvement in DSC supported the validity of the DIR method.

Virtual phantom analysis is another method used to evaluate the appropriateness of the DIR method. We assumed that deformation of the pseudo-rectum by faeces or gas increased only concentrically around the *z*-axis because the rectum is fixed to the retroperitoneum, unlike the sigmoid colon. As illustrated in Fig. 5, a strong correlation between the DIR-based phantom D_mean_ and the simple phantom D_mean_ was observed, although the DIR method included an uncertainty of ∼8.0%. From these results, we considered that the deformation vector field was accurately calculated.

Based on these verifications, patient analysis showed that the rectal D_2cm_^3^, D_1cm_^3^ and D_0.1cm_^3^ values from the simple addition method were overestimated, on average, by 3.1, 3.7 and 5.5 Gy, respectively, relative to the DIR-based values. This finding indicated that as the volume of the DVH parameters decreased, the differences tended to become larger. In other words, this finding implied that D_2cm_^3^ would be more reliable than D*_x_*_cm_^3^ for smaller volumes when the simple addition method is used. In fact, this is consistent with the finding of a previous report, in which rectal D_2cm_^3^ estimated by adding the DVH D_2cm_^3^ parameters of EBRT and ICBT was found to be most relevant for assessment of rectal bleeding [4].

Research on EBRT and ICBT rectal doses estimated by the DIR method for primary cervical cancer has not been reported previously. Andersen *et al.* reported the cumulative dose of the urinary bladder estimated by the DIR method for cervical cancer [9]. Although their target organ was different from that in our study, they also found that DVH parameter addition provided a good estimate for D_2cm_^3^.

The DIR method used in the present study has the following uncertainties in addition to the limit estimated by virtual phantom analysis: inter- and intra-fraction rectal deformation and LQ model in a high-dose region. Nesvacil *et al.* demonstrated that the rectal D_2cm_^3^ variations (SD) were 21.7% in fractionated cervical cancer brachytherapy [10], which indicated that inter- and intrafraction rectal deformation resulted in the largest factor. The LQ model, which was used to calculate the biologically equivalent doses in 2-Gy fractions, coincides well with the range from 1 to 5 or 6 Gy. However, *in vitro* and *in vivo* experiments have shown that there are only small differences between the theoretical and experimental results in high-dose regions. It is difficult to give a specific dose per fraction using the simple LQ model, because extrapolations beyond 5–6 Gy per fraction are likely to lack clinically useful precision [11]. Therefore, when the prescription dose of ICBT was beyond 6 Gy, the cumulative rectal dose extrapolated by the LQ model includes imprecision. In addition to the above-mentioned various uncertainties, it should be noted that the DIR method has an uncertainty of 8%, as shown in the virtual phantom analysis.

To date, there have been a few reports on the use of the DIR method for determination of the total dose delivered to the bladder by ICBT for cervical cancer. However, there have been no reports on the use of the DIR method for determination of the cumulative ICBT and EBRT rectal dose for cervical cancer. Therefore, our study provides results not previously reported. The DIR method may enable the use of multiple treatment planning CT imaging to more accurately estimate the total dose of radical radiotherapy for uterine cervical cancer. We concluded that the difference between simple rectal DVH parameter addition and DIR-based cumulative rectal doses increased when the volume of the DVH parameters decreased. The clinical impact of the difference between the simple rectal DVH parameter addition method and the DIR-based method warrants further investigation.

## SUPPLEMENTARY DATA

Supplementary data are available at the *Journal of Radiation Research* online.

## FUNDING

Funding to pay the Open Access publication charges for this article was provided by the Japan Society for the Promotion of Science (JSPS) KAKENHI Grant No. 25861098.

## Supplementary Material

Supplementary Data

## References

[1] Haie-Meder C Pötter R Van Limbergen E Recommendations from Gynaecological (GYN) GEC-ESTRO Working Group (I): concepts and terms in 3D image based 3D treatment planning in cervix cancer brachytherapy with emphasis on MRI assessment of GTV and CTV. Radiother Oncol 2005;74:235–45. 15763303 10.1016/j.radonc.2004.12.015

[2] Pötter R Haie-Meder C Van Limbergen E Recommendations from gynaecological (GYN) GEC ESTRO working group (II): concepts and terms in 3D image-based treatment planning in cervix cancer brachytherapy—3D dose volume parameters and aspects of 3D image-based anatomy, radiation physics, radiobiology. Radiother Oncol 2006;78:67–77. 16403584 10.1016/j.radonc.2005.11.014

[3] Dimopoulos JCA Petrow P Tanderup K Recommendations from Gynaecological (GYN) GEC-ESTRO Working Group (IV): basic principles and parameters for MR imaging within the frame of image based adaptive cervix cancer brachytherapy. Radiother Oncol 2012;103:113–22. 22296748 10.1016/j.radonc.2011.12.024 PMC3336085

[4] Georg P Lang S Dimopoulos JCA Dose–volume histogram parameters and late side effects in magnetic resonance image-guided adaptive cervical cancer brachytherapy. Int J Radiat Oncol Biol Phys 2011;79:356–62. 20385450 10.1016/j.ijrobp.2009.11.002

[5] Isohashi F Yoshioka Y Koizumi M Rectal dose and source strength of the high-dose-rate iridium-192 both affect late rectal bleeding after intracavitary radiation therapy for uterine cervical carcinoma. Int J Radiat Oncol 2010;77:758–64. 10.1016/j.ijrobp.2009.05.030 19836150

[6] Fowler JF The linear-quadratic formula and progress in fractionated radiotherapy. Br J Radiol 1989;62:679–94. 2670032 10.1259/0007-1285-62-740-679

[7] Akino Y Yoshioka Y Fukuda S Estimation of rectal dose using daily megavoltage cone-beam computed tomography and deformable image registration. Int J Radiat Oncol Biol Phys 2013;87:602–8. 24074934 10.1016/j.ijrobp.2013.06.2054

[8] Zou KH Warfield SK Bharatha A Statistical validation of image segmentation quality based on a spatial overlap index. Acad Radiol 2004;11:178–89. 14974593 10.1016/S1076-6332(03)00671-8 PMC1415224

[9] Andersen ES Noe KØ Sørensen TS Simple DVH parameter addition as compared to deformable registration for bladder dose accumulation in cervix cancer brachytherapy. Radiother Oncol 2013;107:52–7. 23490266 10.1016/j.radonc.2013.01.013

[10] Nesvacil N Tanderup K Hellebust TP A multicentre comparison of the dosimetric impact of inter- and intra-fractional anatomical variations in fractionated cervix cancer brachytherapy. Radiother Oncol 2013;107:20–5. 23602372 10.1016/j.radonc.2013.01.012 PMC3675683

[11] Joiner M Bentzen SM Basic Clinical Radiobiology. 4th edn, London: Hodder Arnold, 2009, 116–7.

