# Supplementary material for: Estimation of the total rectal dose of radical external beam and intracavitary radiotherapy for uterine cervical cancer using the deformable image registration method
Source: J Radiat Res. 2015 Feb 11;56(3):546–52. doi: 10.1093/jrr/rru127 (PMC4426921; doi:10.1093/jrr/rru127)
Supplement: Supplementary Data [file supp_56_3_546__index.html]

Estimation of the total rectal dose of radical external beam and intracavitary radiotherapy for uterine cervical cancer using the deformable image registration method — Estimation of the total rectal dose of radical external beam and intracavitary radiotherapy for uterine cervical cancer using the deformable image registration method — Supplementary Data 

# Estimation of the total rectal dose of radical external beam and intracavitary radiotherapy for uterine cervical cancer using the deformable image registration method

## Supplementary Data

Supplementary Data

**Files in this Data Supplement:**

- Supplementary Data - Docx file
- Supplementary Table - pptx file
