# Supplementary material for: Estimation of the total rectal dose of radical external beam and intracavitary radiotherapy for uterine cervical cancer using the deformable image registration method
Source: J Radiat Res. 2015 Feb 11;56(3):546–52. doi: 10.1093/jrr/rru127 (PMC4426921; doi:10.1093/jrr/rru127)
Supplement: Supplementary Data [file supp_rru127_rru127supp.docx]

Supplymentary table The pattern of source arrangement of intracavitary brachytherapy.

The number above was given every 2.5mm when number one was defined as a point 7mm from the tip of the applicator.
