# Supplementary material for: Estimation of the total rectal dose of radical external beam and intracavitary radiotherapy for uterine cervical cancer using the deformable image registration method
Source: J Radiat Res. 2015 Feb 11;56(3):546–52. doi: 10.1093/jrr/rru127 (PMC4426921; doi:10.1093/jrr/rru127)
Supplement: Supplementary Data [file supp_rru127_rru127supp_table.pptx]

## Slide 1
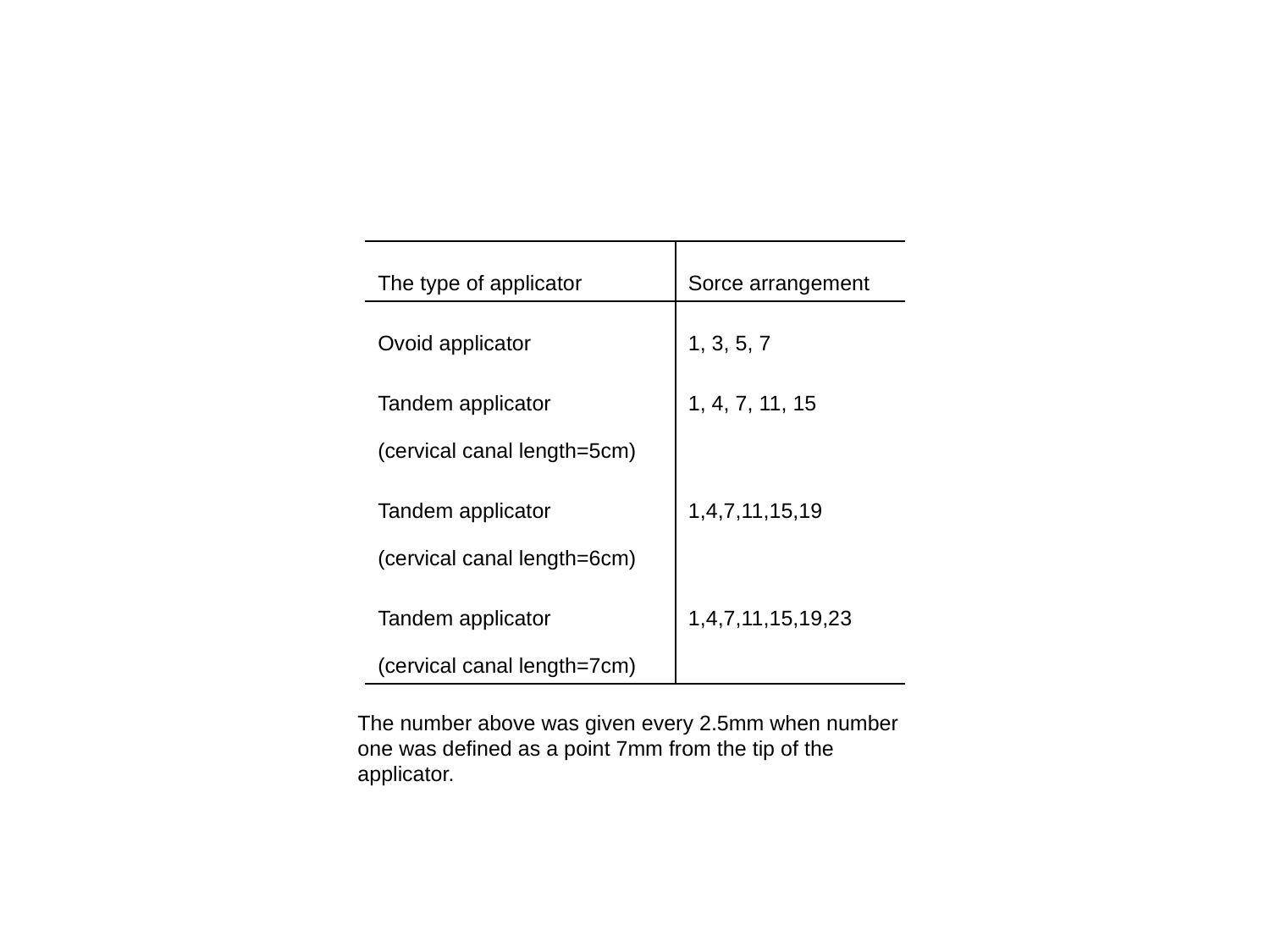

| The type of applicator | Sorce arrangement |
| --- | --- |
| Ovoid applicator | 1, 3, 5, 7 |
| Tandem applicator (cervical canal length=5cm) | 1, 4, 7, 11, 15 |
| Tandem applicator (cervical canal length=6cm) | 1,4,7,11,15,19 |
| Tandem applicator (cervical canal length=7cm) | 1,4,7,11,15,19,23 |
The number above was given every 2.5mm when number one was defined as a point 7mm from the tip of the applicator.
